# Clinical phenotype and molecular characteristics of carbapenem-resistant hypervirulent *Klebsiella pneumoniae* causing multi-site infection

**DOI:** 10.3389/fmicb.2026.1771186

**Published:** 2026-03-04

**Authors:** Hengzhong Lun, Fenfen Liu, Jing Su, Meijie Jiang

**Affiliations:** 1 Department of Laboratory Medicine, The Affiliated Tai’an City Central Hospital of Qingdao University, Tai’an, China; 2 Department of Nephrology, The Affiliated Tai’an City Central Hospital of Qingdao University, Tai’an, China; 3 Department of Geriatric Cardiovascular, The Affiliated Tai’an City Central Hospital of Qingdao University, Tai’an, China

**Keywords:** carbapenem-resistant *Klebsiella pneumoniae*, drug resistance genes, hypervirulent *Klebsiella pneumoniae*, multiple site infection, virulence genes

## Abstract

**Objective:**

To analyze the clinical phenotype and molecular characteristics of three Carbapenem-resistant hypervirulent *Klebsiella pneumoniae* (CR-hvKp) strains isolated from different sites of the same patient, providing an experimental basis for clinical anti-infection treatment and nosocomial infection prevention and control.

**Methods:**

The collected strains were tested for antimicrobial susceptibility using an automatic drug sensitivity analyzer. Whole-genome sequencing was performed to analyze the presence of resistance and virulence genes and to determine the classification and homology of the three strains.

**Results:**

The three *K. pneumoniae* strains were classified as ST11/K47/O13. Whole-genome sequencing revealed that all strains carried the *KPC*-type carbapenemase gene, and the high-virulence genes, rmpA2, *iroB* and *iutA*, along with three plasmids. Antimicrobial susceptibility testing showed that all strains were resistant to carbapenems, including imipenem and meropenem.

**Conclusion:**

The three clinically isolated *K. pneumoniae* strains were highly virulent and carbapenem-resistant, all carrying the *KPC* resistance gene. They caused multi-site infections through hematogenous dissemination. These findings highlight the need for heightened clinical vigilance and strengthened monitoring, prevention, and control of drug-resistant infections.

## 1 Introduction

*Klebsiella pneumoniae* is a common opportunistic pathogen in clinical settings, causing pneumonia, bloodstream infections, urinary tract infections, and other diseases, particularly in immunocompromised populations (Zhewei et al., 2025; Yahui et al., 2023). In recent years, the extensive use of antimicrobial agents has led to a rising prevalence of carbapenem-resistant *K. pneumoniae* (CRKP), posing a severe global public health challenge (Li et al., 2023). More importantly, genetic recombination between traditional hypervirulent *Klebsiella pneumoniae* (hvKp) and CRKP has produced carbapenem-resistant, hypervirulent *K. pneumoniae*, characterized by both high drug resistance and high virulence. This combination significantly limits clinical treatment options and increases patient mortality (Thomas and Candace, 2019). Meanwhile, CR-hvKP is prone to transmission through multiple pathways in high-risk settings such as intensive care unit (ICU), posing formidable challenges to nosocomial infection prevention and control (Yanjun et al., 2025). Existing detection methods are unable to achieve rapid identification, which readily leads to the spread of outbreaks.

In this study, CR-hvKP was simultaneously detected in the blood, gallbladder aspirate and fecal samples of a patient, and such clinical cases are extremely rare in clinical practice. By analyzing the phenotypic and molecular characteristics of isolates obtained from multiple sites of the same patient, this study can provide critical experimental evidence for the rapid diagnosis, precise treatment, and infection prevention and control of CR-hvKP, thus holding substantial clinical application value. Furthermore, The ST11 clone described in this study was found to be identical to the major epidemic clone of CR-hvKP circulating in northern China; this epidemiological representativeness underscores the clinical and public health implications of the present case study.

## 2 Materials and methods

### 2.1 Bacterial strains

The strains were isolated from blood, gallbladder puncture fluid, and feces of a 58-year-old man admitted to the ICU of a teaching hospital in Shandong Province, China. The isolates were identified as *K. pneumoniae* using Autofms1000 automatic microbial mass spectrometer (Autobio). This study was approved by the Ethics Committee of Tai’an Central Hospital (no. 2024-06-76) and conducted according to approved guidelines. The *K. pneumoniae* isolated from blood was designated Blood-KP, from feces as Faeces-KP, and from gallbladder puncture fluid as 2,302,278,006.

### 2.2 Drug sensitivity test

Following CLSI 2023 standards, the MicroScan WalkAway 96 Plus Automated Microbial Susceptibility System (Siemens Healthineers USA) was used to assess susceptibility to over 20 commonly used clinical antibiotics. The automated microbial susceptibility system employs the broth microdilution method for antimicrobial susceptibility testing. The minimum inhibitory concentrations (MICs) of ceftazidime-avibactam were determined through an E-test (Wenzhou Kangtai Biotechnology Co., Ltd.). *Escherichia coli* ATCC 25922 served as the quality control strain.

### 2.3 Determination of high viscosity phenotype

The hyperviscous phenotype was assessed using the string test. Strains were incubated overnight at 37 °C on blood agar plates. Colonies were stretched with an inoculation loop, and adhesive strings longer than 5 mm were considered positive (Naoki et al., 2024).

### 2.4 Carbapenemase phenotype detection

The carbapenemase phenotype was detected using a carbapenemase detection kit (Beijing Kingsoft Technology Development Co., Ltd.), which qualitatively identified the *in vitro* production of *KPC*, *NDM*, IMP, *VIM*, and *OXA*-48 carbapenemases by the cultured bacteria.

### 2.5 Mouse death test

To assess the virulence of the three *K. pneumoniae* isolates, pathogen-free, 6- to 8-week-old C57BL/6 mice [Shandong Pengyue Experimental Animal Science and Technology Co., Ltd. (license no. SCXK (Shandong) 2022–000)] were used. Ten mice per group were infected intraperitoneally with 0.1 mL of bacterial suspension at a concentration of 10^5^ CFU in 0.9% NaCl. *K. pneumoniae* ATCC 13883 served as the control. After infection, the mice were observed twice daily to record their mental status, food and water intake, mobility, body weight changes, and mortality. Mice were identified as exhibiting positive infection symptoms if they presented with obvious lethargy, anorexia, or a sudden body weight loss of ≥10%. Symptoms and mortality rates were observed for 10 days.

### 2.6 Whole genome sequencing and bioinformatics analysis

The genomic sequence of strain 2,302,278,006 was obtained by Shanghai Lingen Biotechnology Co., Ltd. Whole-genome sequencing was performed using Pacific Biosciences Sequel II technology (PacBio). Raw PacBio reads were converted to fasta format with Samtools Fasta. we used unicycler to perform genome assembly with default parameters and received the optimal results of the assembly. Gene models were blastp against different functional types of databases to do functional annotation by blastp module, including non-redundant database, SwissProt, KEGG, COG, CARD, VFDB, etc. Single nucleotide polymorphism (SNP) detection was conducted using Snippy 4.6.0, and the resulting SNP data were summarized into a genetic distance matrix with snp-dists 0.8.2 for strain homology analysis. The genome sequence was further analyzed via the Pathogenwatch platform, which was used to perform sequence typing.

## 3 Results

### 3.1 Clinical features of patients

The patient was admitted to the Department of Gastroenterology with cough and expectoration and was later transferred to the ICU due to respiratory and heart failure. He received life-saving and symptomatic treatment in the ICU. During hospitalization, ultrasound-guided percutaneous cholecystostomy was performed due to biliary obstruction, with an indwelling gallbladder catheter. On February 19, 2023, his body temperature suddenly rose to 38.9 °C, with leukocyte countat 16.63 × 10^9^/L and procalcitonin at 5.42 ng/mL. *K. pneumoniae* was cultured from his blood on February 23, 2023, and subsequently isolated from his feces and gallbladder puncture fluid on February 27, 2023. Prior to the onset of fever, *Pseudomonas aeruginosa* and *Acinetobacter baumannii* were detected positive in his sputum culture, and he was clinically administered meropenem combined with tigecycline for anti-infection therapy. Subsequent blood culture identified carbapenem-resistant *Klebsiella pneumoniae*, after which the therapeutic regimen was adjusted to ceftazidime-avibactam at a dose of 1.25 g once daily, with a total treatment course of 20 days. Following the aforementioned treatment, the patient’s infection-related symptoms were gradually alleviated, and his body temperature and leukocyte count gradually returned to normal.

### 3.2 Drug sensitivity test results

Drug susceptibility testing of the three *K. pneumoniae* strains showed highly consistent results. All strains were resistant to 17 commonly used clinical antibiotics, with sensitivity only to aminoglycosides, trimethoprim-sulfamethoxazole, tigecycline, and ceftazidime-avibactam. All three strains were resistant to carbapenems, including imipenem and meropenem, confirming them as carbapenem-resistant *K. pneumoniae*.

### 3.3 Virulence of three *Klebsiella pneumoniae* strains

**Figure 1:**
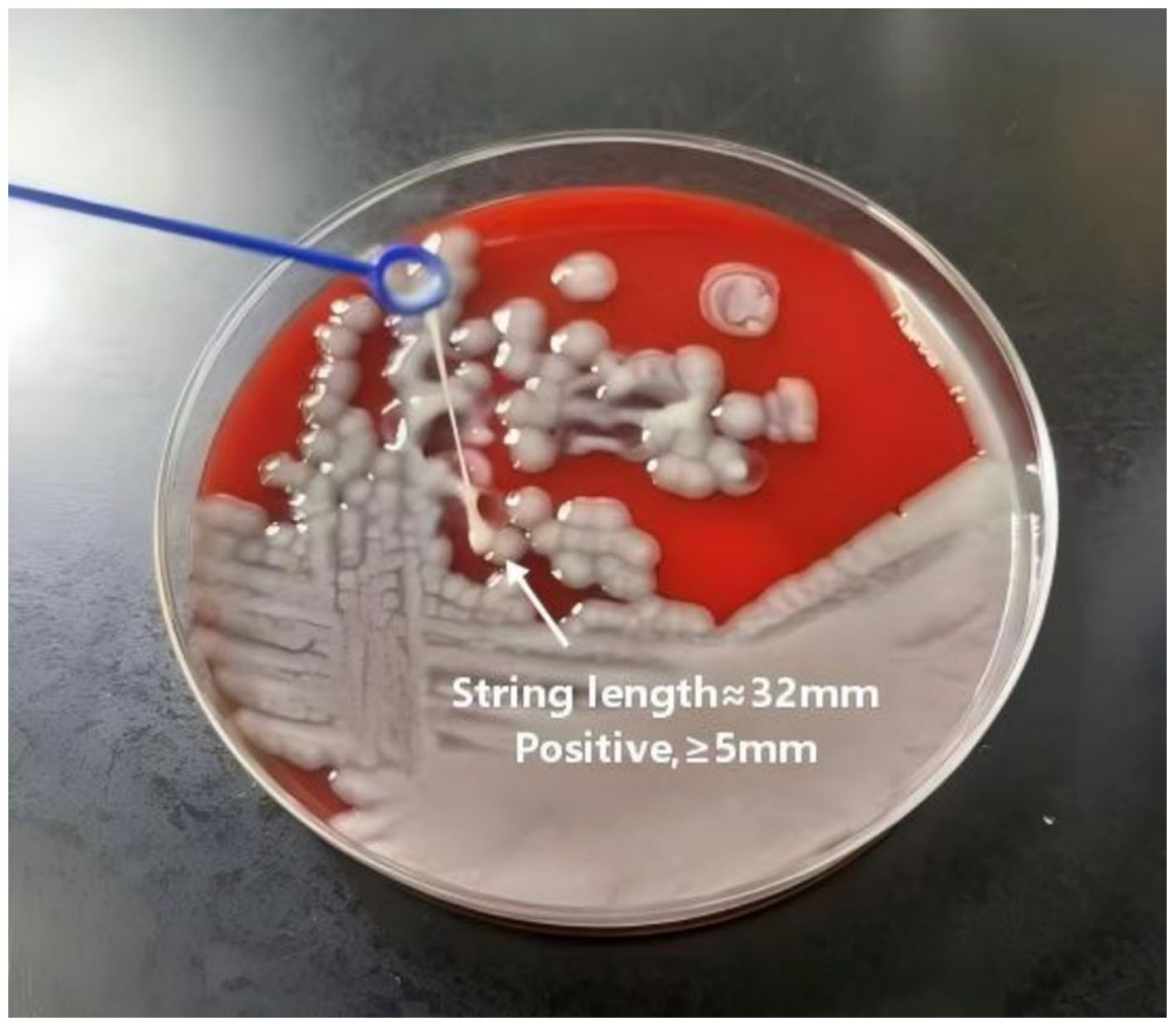
String test of *K. pneumoniae*.

**Figure 2:**
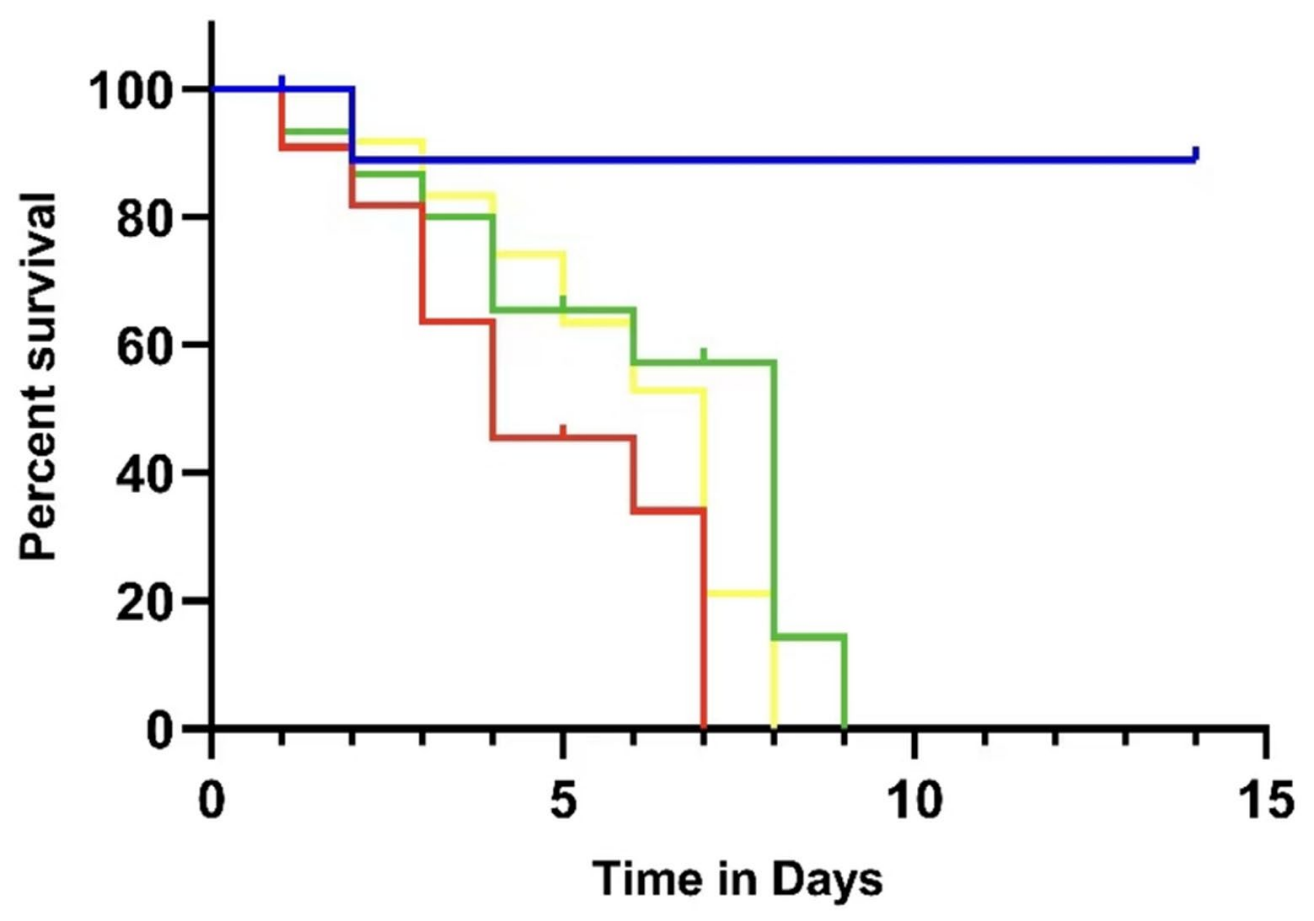
Survival rate of mice infected with three hypervirulent *K. pneumoniae* isolates. Red represents Faeces-KP, green represents Blood-KP, yellow represents 2,302,278,006, and blue represents ATCC 13883.

The hypermucoviscous phenotype was assessed using the string test. Bacterial colonies on agar plates were stretched with an inoculation loop, and all three isolates produced viscous strings exceeding 5 mm, indicating a positive result (Figure 1). In mouse infection experiments, the three *K. pneumoniae* isolates exhibited high virulence comparable to *K. pneumoniae* ATCC 13883. The mean mortality on days 3 and 5 was 20 and 40%, respectively, and by day 8, all mice infected with the clinical isolates had died. In contrast, the mortality rate for mice infected with ATCC 13883 was only 10% (Figure 2).

### 3.4 Carbapenemase phenotype

**Figure 3:**
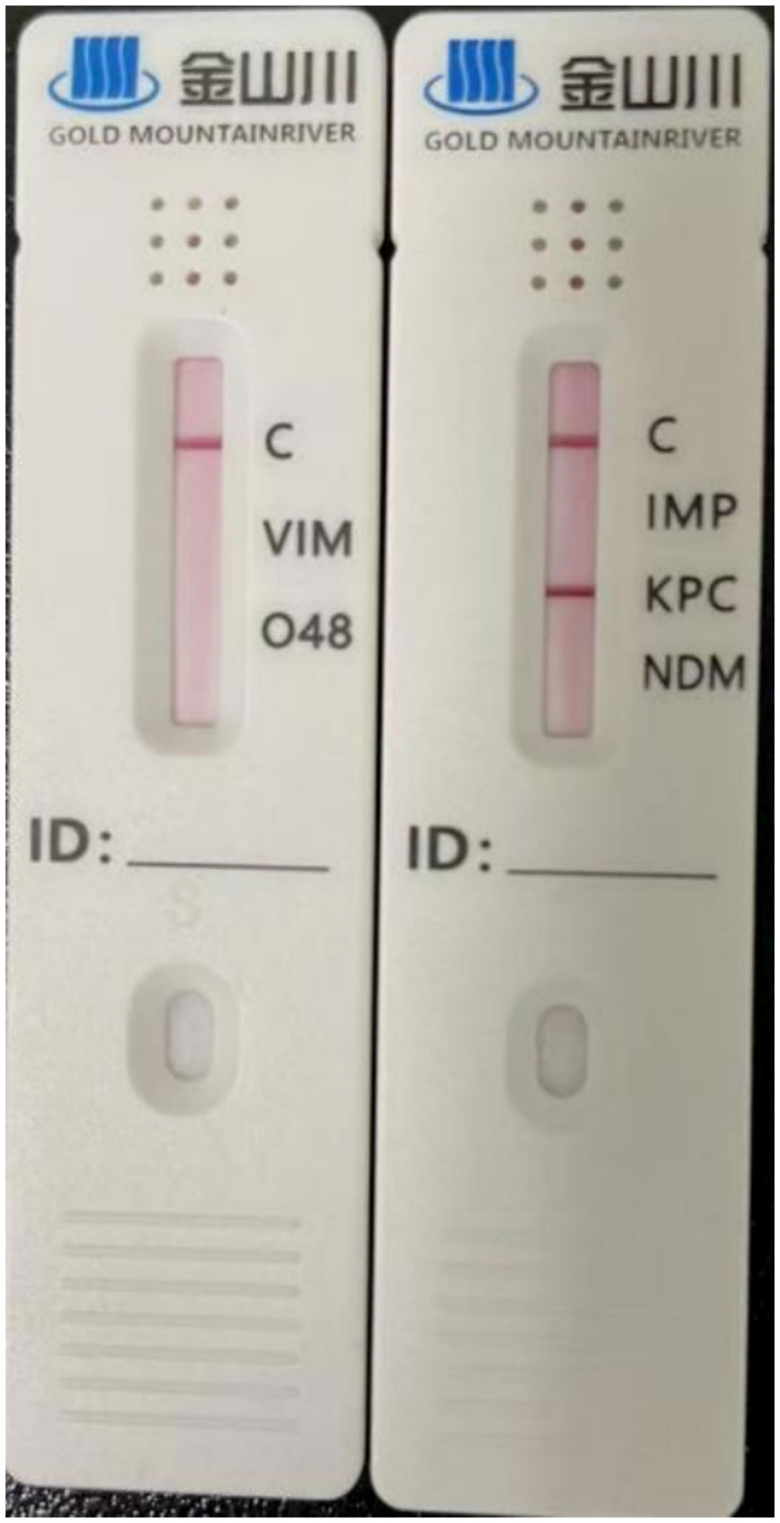
Detection results of carbapenemase phenotype.

Carbapenemases are *β*-lactamases capable of hydrolyzing at least one carbapenem antibiotic. They are classified as Class A, B, or D enzymes. Class B enzymes are metallo-β-lactamases (e.g., IMP, *VIM*, and *NDM*), while Class A (e.g., *KPC*) and Class D (e.g., *OXA*) are serine β-lactamases. According to the kit instructions, all three *K. pneumoniae* strains produced *KPC*-type carbapenemase (Figure 3).

### 3.5 Basic characteristics of *Klebsiella pneumoniae* 2,302,278,006

**Figure 4:**
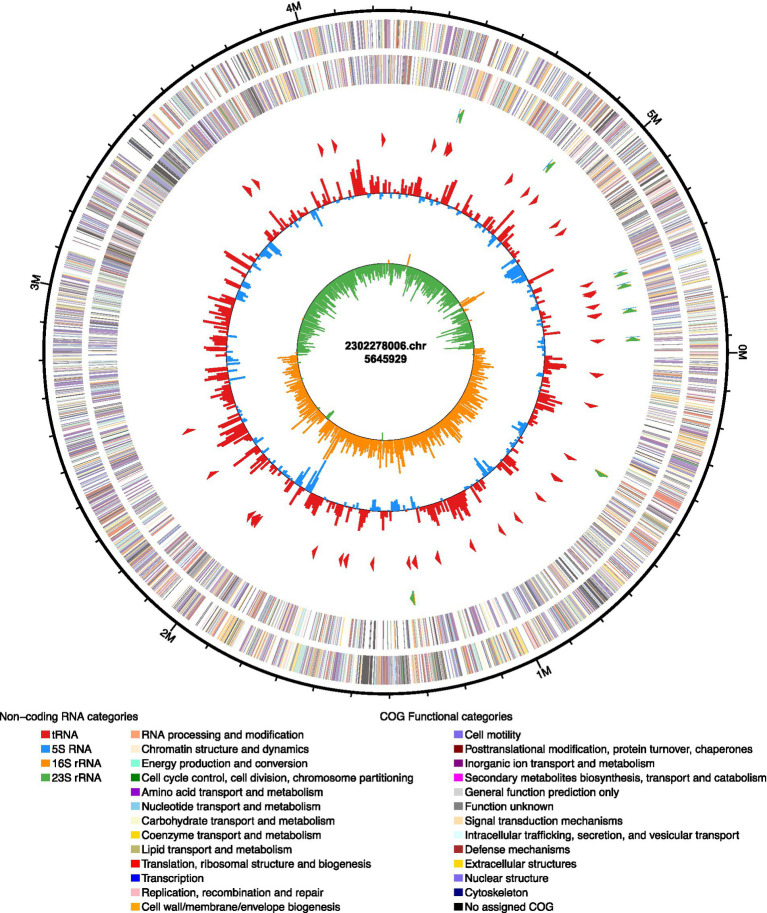
Strain COG functional annotation.

**Figure 5:**
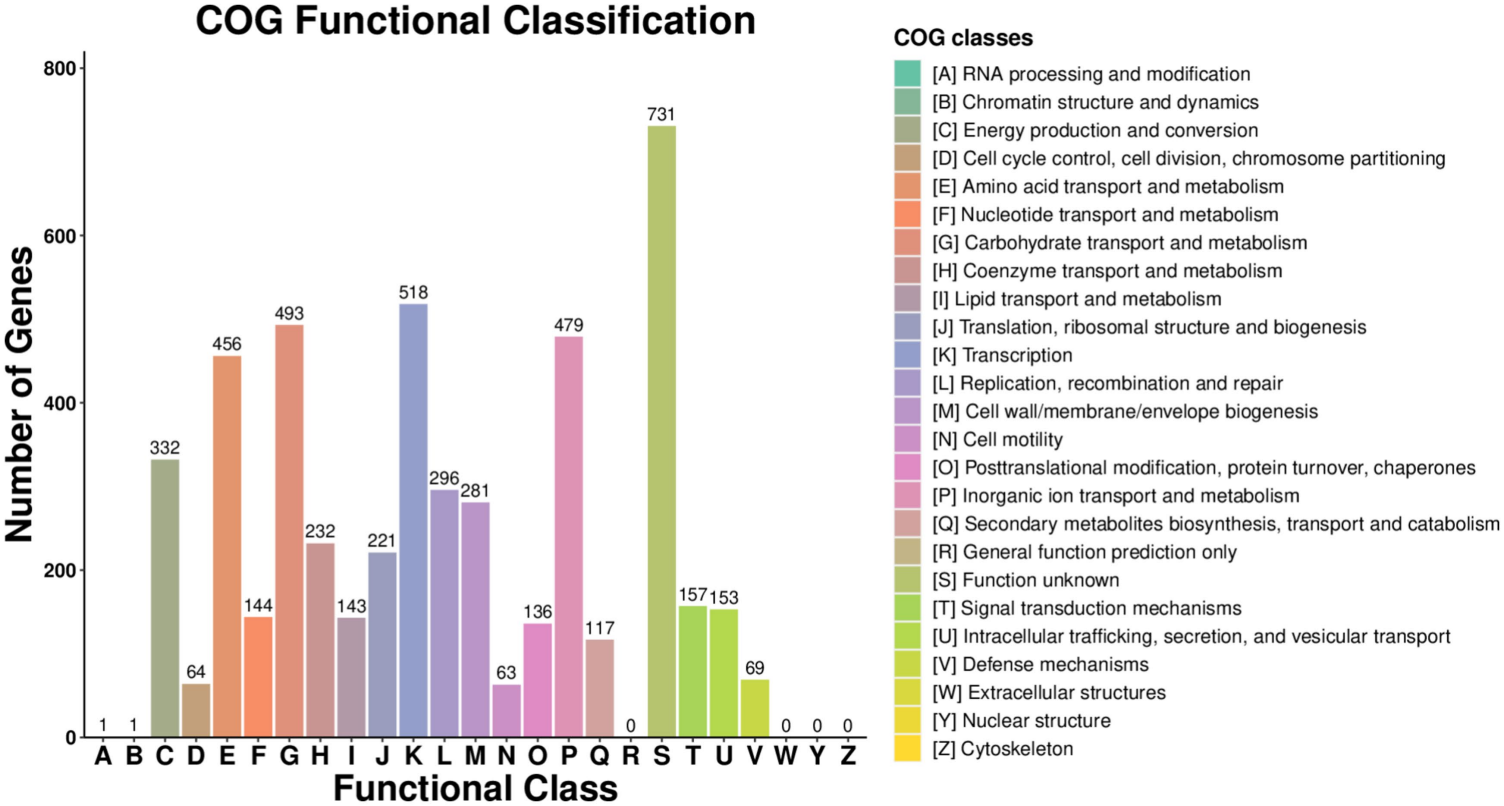
COG functional classification of strain.

Whole-genome sequencing of strain 2,302,278,006 revealed a genome comprising one chromosome and three plasmids. The chromosomal genome was 5,645,929 bp in size, with a GC content of 56.82%. A total of 5,722 proteins were annotated, including 2,717 in the GO database and 3,614 in the KEGG database. COG functional annotation showed that 518 genes were involved in transcription, 493 in carbohydrate transport and metabolism, 479 in cellular transport and metabolism, and 456 in amino acid transport, with fewer genes associated with other functions (Figures 4, 5).

### 3.6 Strain typing and homology

Multilocus sequence typing (MLST) revealed that all three *K. pneumoniae* strains belonged to sequence type 11 (ST11), with the allele profile of seven housekeeping genes (*gapA, infB, mdh, pgi, phoE, rpoB, tonB*) as 3–3–1-1-1-4. Capsular typing identified the K locus as KL47, predicting capsular type K47, while O-antigen typing identified the O locus as OL13, predicting O type O13. SNP analysis showed a distance of 2 between 2,302,278,006 and Blood-KP, 1 between 2,302,278,006 and Faeces-KP, and 3 between Blood-KP and Faeces-KP. Given the ST11 clone threshold (≤16 SNPs), the SNP distances ≤3indicated that the three strains belonged to the same clone (Table 1).

**Table 1:** SNP analysis of three strains of bacteria.

SNP-dists 0.8.2	2,302,278,006	Blood-KP	Faeces-KP
2,302,278,006	0	2	1
Blood-KP	2	0	3
Faeces-KP	1	3	0

### 3.7 Drug resistance genes

**Figure 6:**
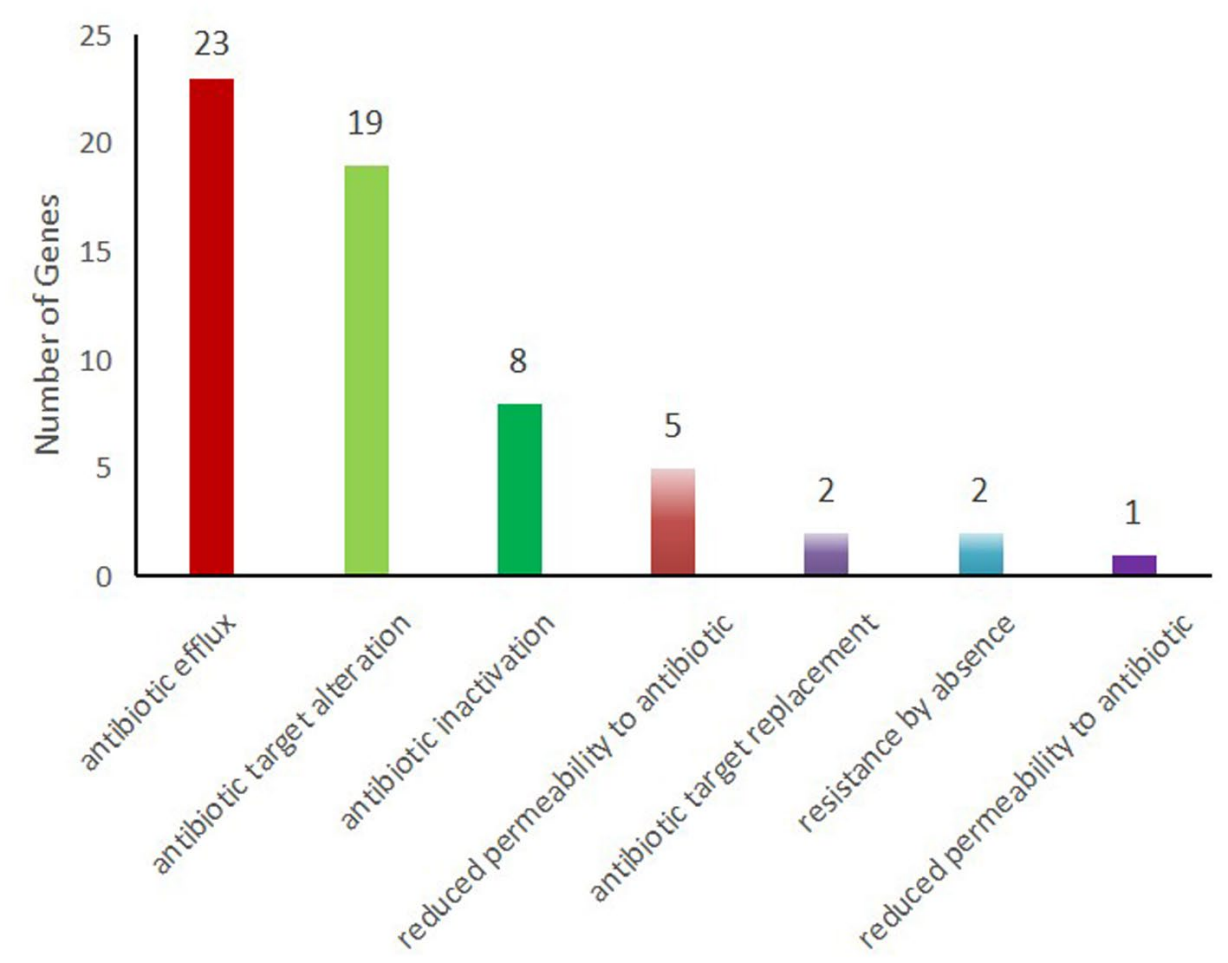
Resistance mechanisms associated with resistance genes. The ordinate represents the number of resistance genes, and the abscissa represents the corresponding resistance mechanisms.

Using a sequence identity threshold of ≥90%, 49 resistance-related genes were detected in *K. pneumoniae* 2,302,278,006 via the CARD database (Table 2). The core resistance genes included *KPC-2* (carbapenem resistance), CTX-M-65 (third-generation cephalosporin resistance), *oqxA* and *oqxB* (quinolone resistance), *sul1* (sulfonamide resistance), and *FosA3* (fosfomycin resistance). Sequence alignment showed 100% identity for the *KPC-2* gene, and no variants such as *bla*KPC-135 or *bla*KPC-144, nor other carbapenemase genes (*NDM*, *OXA*-48, *VIM*) were detected. This indicated that carbapenem resistance in this strain was mediated solely by *KPC-2*, consistent with the carbapenemase phenotyping results. Of the 49 resistance genes, 23 were involved in antibiotic efflux, 19 in antibiotic target substitution, and 8 in antibiotic inactivation. Some genes contributed to multiple resistance mechanisms (Figure 6).

**Table 2:** Drug resistance genes.

Identity%	Gene
100	*oqxB*, *oqxA*, *Klebsiella pneumoniae kpnF*, *sul1*, *Klebsiella mutant phoP* conferring antibiotic resistance to colistin, *Klebsiella pneumoniae OmpK35*, *Klebsiella pneumoniae acrR* with mutation conferring multidrug antibiotic resistance, *bla*KPC-2, *fosA3*, *bla*CTX-M-65
96–99.99	*Klebsiella pneumoniae KpnG*, *SHV-70*, *SHV-64*, *Klebsiella pneumoniae KpnE*, *CRP*, *aadA3*, *Escherichia coli EF-Tu* mutants conferring resistance to pulvomycin, *Escherichia coli EF-Tu* mutants conferring resistance to kirromycin, *Escherichia coli rpoB* mutants conferring resistance to rifampicin, *catII* from *Escherichia coli K-12 cat*, *Klebsiella pneumoniae acrR* with mutation conferring multidrug antibiotic resistance, F*osA6*, *Salmonella enterica gyrA* with mutation conferring resistance to triclosan
90–95.99	*Escherichia coli PtsI* with mutation conferring resistance to fosfomycin, *Escherichia coli UhpT* with mutation conferring resistance to fosfomycin, *Escherichia coli gyrB* conferring resistance to aminocoumarin, *Klebsiella pneumoniae acrA*, *cpxA*, *Escherichia coli parC* conferring resistance to fluoroquinolone, *Escherichia coli parE* conferring resistance to fluoroquinolones, *Klebsiella pneumoniae OmpK37*, *H-ns*, *emrB*, *Escherichia coli UhpA* with mutation conferring resistance to fosfomycin, antibiotic-resistant *fabI*, *Escherichia coli GlpT* with mutation conferring resistance to fosfomycin, *Escherichia coli CyaA* with mutation conferring resistance to fosfomycin, *msbA*, *marA, Klebsiella pneumoniae OmpK36*, *Escherichia coli murA* with mutation conferring resistance to fosfomycin, *emrR*, *baeR*, *acrB*, *mdtC*, *acrD*, Salmonella serovars *soxS* with mutation conferring antibiotic resistance, *Escherichia coli soxR* with mutation conferring antibiotic resistance, *mdtB*

### 3.8 Virulence genes

**Figure 7:**
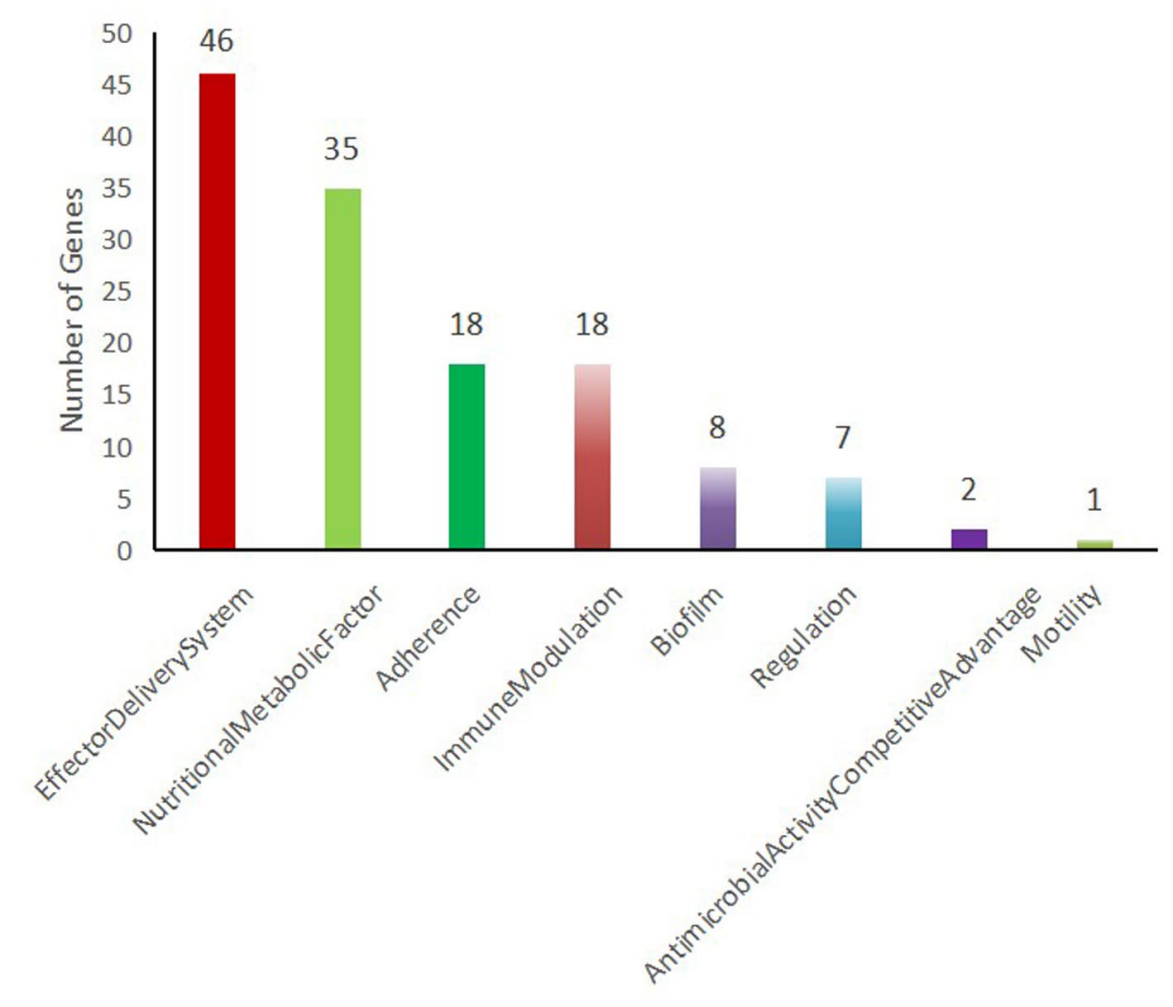
Functional classification of virulence genes. The ordinate represents the number of genes, and the abscissa represents their associated functions.

Using a sequence identity threshold of ≥80%, 135 virulence-related genes were detected in *K. pneumoniae* 2,302,278,006 via the VFDB database (Table 3). Biomarkers such as *peg*-344, *iroB*, *iutA*, *rmpA*, and *rmpA2* are highly accurate (diagnostic accuracy >0.95) for identifying hvKp. In this strain, high-virulence genes *rmpA2*, *iroB*, and *iutA* were detected. Functional annotation showed that 46 of the 135 virulence genes were associated with the effector delivery system, 35 with nutrient acquisition and metabolism, 18 with adherence, and 18 with immunomodulation (Figure 7).

**Table 3:** Virulence genes.

Identity%	Gene
100	*fimK*, *fimH*, *fimF*, *fimC*, *fimI*, *fimA*, *fimB*, *mrkA*, *mrkB*, *mrkC*, *mrkD*, *mrkF*, *mrkJ*, *mrkH*, *clpV*, *rcsB*, *galF*, *KPHS_35550*, *ugd*, *rfbC*, *fyuA*/*psn*, *ybtE*, *ybtT*, *ybtU*, *irp1*, *ybtA*, *ybtP*, *ybtQ*, *ybtX*, *ybtS*, *rcsA*, *impJ*, *impG*, *impH*, *sciN*, *impF*, *impA*, *A79E_RS10215*, *N559_RS10440*, *sciN*/*tssJ*, *tssG*, *tssF*, *impA*/*tssA*, *icmF*/*tssM*, *KPHS_23120*, *tli1*, *KPHS_23050*, *clpV*/*tssH*, *hcp*/*tssD*, *ompA*, *vasE*/*tssK*, *vipB*/*tssC*, *vipA*/*tssB*, *iroN*, *iutA*, *entA*, *entB*, *entE*, *entC*, *entS*, *fepD*, *fepC*, *entF*, *fes*, *fepA*, *entD*, *acrA*, *gspG*, *iucA*, *iucB*, *iucC*, *iucD*
96–99.99	*fimD*, *irp2*, *vgrG*/*tssI*, *acrB*, *gndA*, *KPHS_35560*, *KPHS_35570*, *KPN_RS13395*, *iroE*, *KPHS_23130*, *fepB*, *fepG*, *KPN_RS13400*, *KPN_RS13390*, *dotU*/*tssL*, *fimE*, *fimG*, *mrkI*, *KPHS_35580*, *gspI*, *rmpA2*, *KPN2242_RS16315*, *KPHS_35700*, *icmF*, *KPR_RS09045*, *rpoS*, *dotU*, *KPHS_32780*, *gspE*, *KPK_RS21755*, *gspS*, *gspD*, *KPN_RS13385*, *gspJ*, *gspF*, *gspK*, *KPN2242_RS16305*
91–95.99	*yagZ*/*ecpA*, *fur*, *tle1*, *yagW*/*ecpD*, *gspH*, *yagX*/*ecpC*, *phoP*, *gspL*, *ykgK*/*ecpR*
86–90.99	*gspB*, *KPK_RS21750*, *yagY*/*ecpB*, *wbaP*, *tcyJ*, *yagV*/*ecpE*, *KPK_RS21745*, *hcpA*, *gspC*
81–85.99	*iroB*, *sitC*, *kdsA*, *EC55989_RS17100*, *PMI_RS01110*, *tufA*, *phoQ*, *sitB*

### 3.9 Plasmids

**Figure 8:**
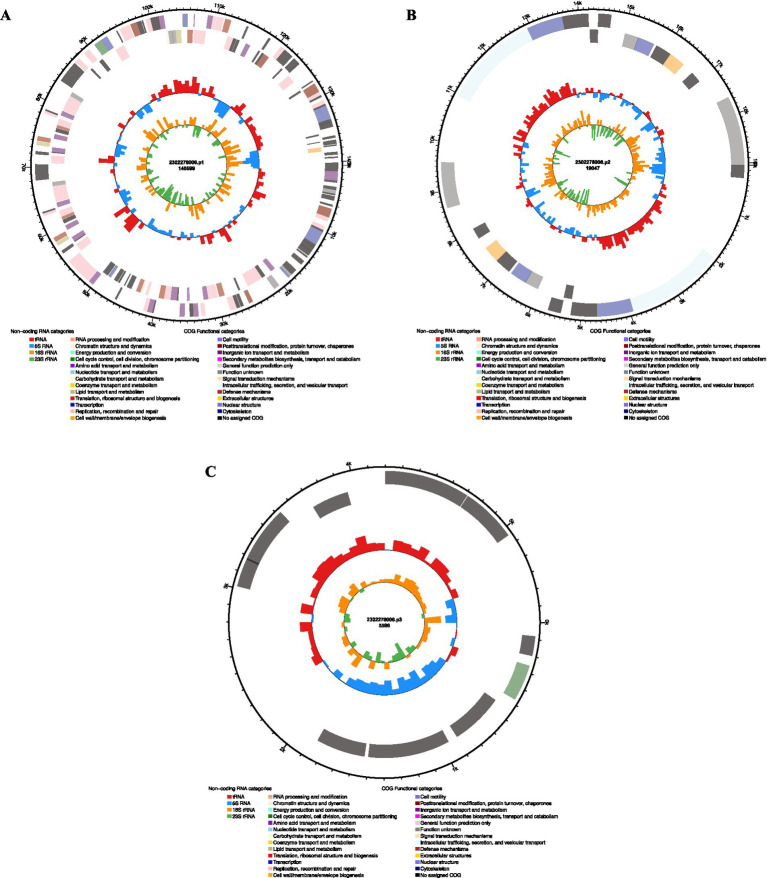
Functional analysis of plasmid COGs: **(A)** Functional analysis of plasmid 1; **(B)** Functional analysis of plasmid 2; **(C)** Functional analysis of plasmid 3.

Whole-genome sequencing of *K. pneumoniae* strain 2,302,278,006 revealed a genome comprising one chromosome and three plasmids. Plasmid 1 was a large plasmid (140,599 bp) with major COG functions including replication, recombination and repair, amino acid transport and metabolism, and posttranslational modification, protein turnover, and chaperones. Plasmid 2 measured 19,047 bp, with COG functions related to cell motility and coenzyme transport and metabolism. Plasmid 3 was a small plasmid (5,596 bp) with COG functions in cell cycle control, cell division, and chromosome partitioning (Figure 8). These three plasmids covered distinct functional roles, which may synergistically contribute to the systemic spread of the bacterium.

## 4 Discussion

In this study, three CR-hvKp strains (ST11/K47/O13) were isolated from blood, gallbladder aspirate, and feces of the same patient. The ST11 clonal population is a dominant epidemic clone of CRKP, often carrying multiple drug resistance and virulence genes (Yuxia et al., 2026). ST11-type CRKP can spread via patient contact, contaminated medical equipment, and transmission by healthcare workers’ hands. ICU patients are at particularly high risk due to multiple invasive procedures and compromised immune function (Yi-Le et al., 2025).

Hypervirulent *Klebsiella pneumoniae* was initially also referred to as hypermucoviscous *Klebsiella pneumoniae*. Therefore, at the early stage of its discovery, laboratories mostly determined the virulence of strains solely based on the length of string formation from single colonies. At present, the gold standard for confirming hvKP strains is animal experimentation, and the mouse median lethal dose (LD50) assay is commonly used to verify strain virulence. In addition, the combined identification of virulence genes including *peg344, iroB, iucA, rmpA, and rmpA2* can accurately distinguish hvKP from classical *Klebsiella pneumoniae*, with an accuracy of over 95% (Thomas et al., 2018). All strains in this study exhibited a hypermucoviscous phenotype, high mouse lethality, and carried core virulence genes *rmpA2, iroB, and iutA,* which confirmed that the isolated strains were hvKP. The synergistic action of these genes underlies the high pathogenicity of CR-hvKp: *rmpA2* regulates capsular polysaccharide synthesis to enhance anti-phagocytic ability, while *iroB* and *iutA* contribute to siderophore synthesis to satisfy iron requirements, collectively promoting bacterial colonization and replication in multiple host organs (Xuemei et al., 2020; Min et al., 2024). Virulence gene analysis also identified core genes such as *fimA* (type 1 fimbrial), *mrkD* (type 3 fimbrial), and *iutA* (aerobactin), indicating strong virulence potential (Parvathy et al., 2023). Type 1 and type 3 pili mediate bacterial adhesion to host epithelial surfaces, enhancing infectivity. Capsular polysaccharides inhibit phagocytosis and enable immune evasion, while iron acquisition systems such as aerobactin and enterobactin support bacterial growth in iron-limited host environments (Fatma et al., 2025). The patient’s history of biliary obstruction and invasive procedures represented high-risk factors for CR-hvKp infection, facilitating bacterial invasion by disrupting mucosal barriers and reducing immune defense (Longyang et al., 2020).

The resistance mechanisms of CRKP primarily include carbapenemase production, porin deletion, and overexpression of drug efflux pumps, with *KPC*-type carbapenemase being the main mechanism (Karen, 2018). In this study, all three *K. pneumoniae* strains carried the *KPC* gene and exhibited high-level resistance to carbapenems, including imipenem and meropenem (MIC > 32 μg/mL). The observed resistance phenotype was highly consistent with the genotype, confirming that *KPC* was the key determinant of carbapenem resistance. Additionally, the three strains carried multiple resistance genes, including *bla*CTX-M-65, *oqxA*, and *oqxB*, conferring resistance to cephalosporins and quinolones and resulting in a typical multidrug-resistant phenotype (Rodríguez-Martínez et al., 2012; Daniel et al., 2025). Although the *sul1* gene can mediate sulfonamide resistance by encoding a variant of dihydropteroate synthase, the strains remained sensitive to trimethoprim-sulfamethoxazole, possibly due to low *sul1* expression (Meenakshi et al., 2023).

Currently, effective treatment options for CR-hvKp infections are limited, with ceftazidime-avibactam, tigecycline and polymyxin commonly used. However, increased usage has led to rising resistance rates. The approval of new antibacterial agents, such as aztreonam-avibactam, has provided alternative options for treating metallo-*β*-lactamase-producing CR-hvKp (Dafna et al., 2020). In this study, after CRKP was isolated from the patient’s blood culture, antimicrobial susceptibility testing indicated that the strain was susceptible to ceftazidime-avibactam. The patient’s condition gradually improved following clinical treatment with this agent, highlighting the importance of timely, susceptibility-guided therapy to improve prognosis.

Studies have shown that the intestinal tract is a key site for CR-hvKp colonization. In this study, since the patient had no obvious gastrointestinal infection symptoms, the strain isolated from feces was considered to be colonization. Colonized strains can enter the bloodstream through damaged mucosa and subsequently disseminate to multiple organs, causing multi-site infections—a transmission pathway particularly common in ICU patients (Qiao-Ling et al., 2019). In this study, the three *K. pneumoniae* strains isolated from feces, blood, and gallbladder puncture fluid were confirmed by SNP analysis to belong to the same clone, suggesting that multi-site infection might occur via intestinal colonization and hematogenous dissemination. Whole-genome sequencing revealed that the strain carried three plasmids with complementary functions, consistent with the integration of resistance and virulence genes on mobile genetic elements via plasmid fusion and horizontal transfer (Binghui et al., 2025). These plasmids contributed to survival and adaptation, facilitated dissemination and energy supply, and regulated proliferation and colonization efficiency. Virulence genes such as *rmpA2*, *fimH*, *mrkD*, and *ybt* were associated with intestinal colonization, mucosal invasion, systemic dissemination, and tissue damage (Ozlem et al., 2021; Dokyun et al., 2018), collectively forming the molecular basis for the sequence: “intestinal colonization → mucosal invasion → hematogenous dissemination → cross-tissue survival.” In addition, biliary tract infection may also serve as the primary infection source. After the bacteria enter the bloodstream and induce bacteremia, they spread to the intestinal tract via biliopancreatic reflux, resulting in bacterial shedding in feces.

## 5 Conclusion

The three *K. pneumoniae* strains isolated in this study exhibited typical multidrug resistance and high virulence potential. Carbapenem resistance was primarily mediated by *KPC*, and the strains carried multiple virulence genes. High genomic homology indicated that the strains belonged to the same clone. Clinically, it is recommended to establish a diagnostic system centered on molecular detection to rapidly identify CR-hvKp by detecting virulence factors, specific ST-types and serotype-encoding genes, and carbapenemase genes, providing a basis for timely clinical diagnosis and treatment.

## Data Availability

The datasets presented in this study can be found in online repositories. The names of the repository/repositories and accession number(s) can be found at: https://www.ncbi.nlm.nih.gov/, SAMN54134050.
